# Bioethics and the soft power of sport: a contemporary perspective

**DOI:** 10.3389/fspor.2025.1683697

**Published:** 2025-10-23

**Authors:** Marina Kamenecka-Usova

**Affiliations:** ^1^ Faculty of Social Sciences, Rīga Stradiņš University, Riga, Latvia; ^2^ Law Department, EKA University of Applied Sciences, Riga, Latvia

**Keywords:** bioethics in sport, ethics of enhancement, global sport governance, performance-enhancing methods, political lobbying, soft power of sport, sports ethics

## Abstract

This article offers a perspective on the political dimensions of bioethics within elite sport, particularly regarding the regulation of performance-enhancing methods. Drawing from historical and contemporary examples, it suggests that bioethical decision-making in sport is often influenced by geopolitical agendas, institutional power relations, and national interests. Focusing on the World Anti-Doping Agency's regulatory process, the article argues that scientific assessments may be shaped or delayed by stakeholder lobbying, soft power strategies, and disparities in global access to biomedical innovation. Rather than providing a comprehensive review, this article reflects the author's viewpoint on how bioethics operates within contested frameworks of legitimacy, equity, and global influence. It concludes that recognizing bioethics as politically embedded is crucial for fostering fairer, more transparent, and globally representative governance in sport.

## 1 Introduction

Ethics can be understood in two ways. First, it refers to well-established standards of right and wrong that outline what individuals ought to do - typically in terms of rights, responsibilities, societal benefit, fairness, or particular virtues. Second, ethics involves the study and continual development of those moral standards. As noted by Velasquez et al. (1), personal feelings, laws, and cultural norms may not always align with what is truly ethical. Therefore, it is essential to regularly examine and evaluate our ethical standards to ensure they are reasonable and well-founded. In this sense, ethics also represents an ongoing commitment to reflect on our moral beliefs and actions, and to work toward ensuring that both we and the institutions we contribute to uphold sound, principled standards (1). Within this intellectual tradition, bioethics emerged as a distinct branch of applied ethics, first articulated by Van Rensselaer Potter in the early 1970s. Potter introduced bioethics as a “bridge to the future” that integrates biological knowledge with human values with the aim of ensuring the survival and flourishing of humankind. From this perspective, bioethics transcends being merely a technical or medical specialty; it is fundamentally a normative discipline, grounded in philosophical reflection yet deeply engaged with practical ethical dilemmas in science, medicine, and society (2). According to Vargas-Mendoza et al. (3) bioethics is defined as the systematic study of human behavior in the context of life and health sciences, examined through the lens of moral values and ethical principles. At its core, bioethics explores the relationship between human biological nature and the broader biological world, aiming to inform policies that promote the well-being of both present and future generations. It is inherently interdisciplinary, drawing on insights from medicine, the humanities, economics, philosophy, politics, and law, and is rooted in an ongoing dialogue between ethical reflection and life sciences (3). By bringing together ethical reflection and policy considerations, bioethics offers a unique and valuable contribution. It raises questions and seeks solutions in ways that often differ from traditional policy analysis. Bioethics engages with some of the most profound aspects of human experience - particularly in moments where life-and-death decisions must be made. Yet, it also plays a role in our everyday lives, promoting health in ethically sound ways while recognizing when the pursuit of health may become excessive in light of other critical societal priorities. Although bioethics as a field is only about 50 years old, it continues to evolve - with much still to learn and much to offer (4). The fundamental principles of bioethics - principlism - first articulated by Tom Beauchamp and James Childress in their seminal work Principles of Biomedical Ethics (5), are four core ethical guidelines widely accepted in healthcare and research involving human subjects:

*Autonomy*: respecting the individual's right to make informed, voluntary decisions about their own life and body without coercion. This includes honoring informed consent and self-determination.

*Beneficence*: the obligation to act in ways that promote the welfare and well-being of others, actively contributing to their health and interests.

*Non-maleficence*: the duty to avoid causing harm to others, encapsulated in the principle “first, do no harm” (primum non nocere). It requires healthcare providers to refrain from actions that could cause unnecessary injury or suffering.

*Justice*: ensuring fairness in the distribution of benefits, risks, and resources, including equitable access to healthcare and fair treatment of all individuals.

Sport, as an integral part of human life, exerts a profound influence on individuals and society, fostering virtues such as perseverance, discipline, and teamwork (6). According to the World Anti-Doping Agency (WADA) Code, the “spirit of sport” embodies the celebration of the human spirit, body, and mind. It represents the core ideals of Olympism and is reflected in the values that sport seeks to uphold and promote. This spirit is ultimately captured in the ethos of “playing true” (7).

Hence, the distinction between recreational and professional sport is crucial when considering ethical issues: while recreational sport emphasizes participation and enjoyment, professional sport is driven by competition, financial incentives, and the pursuit of peak performance. This environment often creates ethical dilemmas, particularly as advances in sports science and medicine offer new ways to enhance performance, sometimes at the expense of fairness and the athlete's well-being. The increasing use of prohibited substances, non-therapeutic gene therapies, and other performance-enhancing methods in professional sport has become a pressing bioethical concern, as these practices can undermine the core values of sport and violate the fundamental principles of bioethics (6). Bioethics, therefore, provides a critical framework for evaluating and addressing these challenges by examining the ethical, social, moral, and legal implications of scientific and medical advances in sport. By promoting ethical decision-making and upholding the integrity of sport, bioethics serves as a bridging science that helps ensure athletic achievement does not come at the cost of fairness, health, or the true spirit of sport (6). Growing literature, including recent contributions from Slivšek et al. (6) and related analyses by Škerbić (8, 9), highlights the need to address sport bioethics as a multidisciplinary field situated at the intersection of medicine, philosophy, law and politics.

A further dimension of bioethics that holds particular significance in the realm of sport is its political dimension. As Gregg (10) argues, bioethics is politics because bioethical questions often cannot be settled by universally accepted rational arguments, but instead require collective decisions about regulation and public policy in the face of persistent disagreement over values. These decisions are inherently political, reflecting varying, socially constructed norms and the need for procedures - like expert committees and democratic deliberation - to reach outcomes that can be regarded as procedurally legitimate, even if they are not universally agreed upon (10).

This article will further examine performance-enhancing methods (PEMs) in sport, with particular focus on performance-enhancing drugs (PEDs) and gene or cell doping. Understanding the complexities and implications of these methods is essential to appreciating their significance - and frequent controversy - in contemporary elite sport. Building on these foundations, the article argues that bioethics in sport must also be examined through the lens of soft power (11). Soft power refers to the ability of states to attract and persuade through cultural, social, and political influence rather than coercion, and in the sporting domain it operates when international success or the hosting of global events becomes a tool of national prestige and influence. The discussion therefore turns to how bioethics in sport intersects with political processes, highlighting the role of international relations in shaping ethical and regulatory frameworks.

## 2 Debate on performance-enhancing methods as a subject of bioethics in sport

The use of *PEDs* in sport is a deeply complex topic at the intersection of science, ethics, and social values. The debate extends far beyond simple notions of cheating, touching on fundamental bioethical principles and the very nature of athletic achievement. This topic has been extensively explored by scholars. For instance, Miah has produced a series of works on the intersection of ethical debates, sport, and PEDs, emphasizing that “the pursuit of human enhancements is complex, contested, and subject to all kinds of ideological impositions” (12).

Key bioethical themes in the PED debate according to Loland (13).

1. *Complexity and Disagreement*. The PED issue is not merely about rule-breaking; it involves significant ethical disagreements among scholars, athletes, and policymakers. Public discourse often oversimplifies PED use as cheating, but ethical justification for bans requires deeper analysis of sport's values and ideals.
2. *Standard Justifications: Fairness and Health*. Arguments for banning PEDs usually focus on fairness (level playing field) and harm to athletes’ health. These justifications are insufficient unless placed within a broader understanding of what sport is meant to represent.
3. *Natural vs. Artificial Performance.* A central theme is the distinction between “natural” and “artificial” enhancement. Natural performance is seen as the product of genetic predispositions and training, while PEDs are viewed as artificial because they bypass natural adaptation and directly alter biological functions.
4. *Normative Structure of Sport.* Sport is built on the principle of fair equality of opportunity: athletes should compete under conditions where success reflects natural talent and effort, not artificial enhancement. Classification systems (by sex, weight, etc.) and standardization of equipment aim to compensate for factors outside athletes’ control, reinforcing meritocracy.
5. *Authenticity and Athlete Responsibility.* Authentic performance is valued as an expression of the interplay between an individual's genetics and environment. PED use challenges the authenticity of sport and diminishes athlete responsibility and agency.
6. *Acceptable vs. Unacceptable Enhancement*. Not all enhancement is treated equally. Technological aids (e.g., altitude tents) that exploit natural adaptation are generally accepted. Pharmaceutical PEDs [e.g., Anabolic steroids, Erythropoietin (EPO)/peptide hormones] that override natural processes are not, as they undermine the connection between effort and achievement.
7. *The Spirit of Sport.* Sport is seen as a moral testing ground for human excellence, dignity, and responsibility. The ban on PEDs is justified not only by fairness and health, but also by the “spirit of sport,” which emphasizes the admirable development of natural talent.
8. *Challenges and Gray Areas*. The concept of “natural” is vague and socially constructed, requiring careful operationalization to avoid discrimination. Ongoing debates include harm reduction and the potential for medically controlled PED use, which would challenge traditional values.
9. *Contextual Exceptions.* In crisis situations (e.g., medical emergencies), PED use may be morally justified, but this does not apply to competitive sport. The dialog on PEDs as a subject of bioethics in sport reveals that bans are best justified by a combination of fairness, health, and the ideal of cultivating natural talent and authentic achievement. PED use fundamentally contradicts the normative structure of sport, which is designed to foster human excellence through natural means (13).

Another highly relevant topic within the field of bioethics and sport, deserving separate attention, is *gene doping or cell doping*. In 2004 gene or cell doping was defined by the WADA as “the non-therapeutic use of genes, genetic elements and/or cells that have the capacity to enhance athletic performance.” (14) WADA has since broadened its language to include any manipulation of genetic material: “The use of nucleic acids or nucleic acid analogues that may alter genome sequences and/ or alter gene expression by any mechanism. This includes but is not limited to gene editing, gene silencing and gene transfer technologies.” (15) Gene doping stems from the principles of gene therapy. However, unlike gene therapy - which involves introducing DNA into the body to restore a function lost due to a damaged or missing gene - gene doping entails the insertion of DNA specifically to enhance athletic performance (16). The essential difference between gene doping and conventional doping is the fact that instead of substances such as anabolics, hormones or blood, genetic material or other substances that modify how gene expression is regulated are introduced into the body (17). Advances in genetics and genomics are increasingly applied not only in the diagnosis and treatment of diseases but also raise the possibility of enhancing human physical capabilities. As per Unal and Unal (18), developments in gene therapy have demonstrated promising clinical outcomes, which have, in turn, intensified concerns about the potential misuse of these technologies in sport. This growing apprehension has fueled the ongoing debate surrounding gene doping. Therapeutic interventions originally designed to treat medical conditions - such as the EPO gene for anemia, the insulin-like growth factor-1 (IGF-1) gene for muscular dystrophy, and the vascular endothelial growth factor (VEGF) gene for peripheral vascular diseases—are among those considered susceptible to misuse for performance enhancement (18).

Back in 2006, Haisma and Hon in their paper on Gene Doping wrote that with the rapid advancement of genetic therapies as a promising area within mainstream medicine, concerns have emerged about the potential misuse of these techniques in the realm of sports. Previous experiences have demonstrated that substances still in the experimental stages of research can enter athletic competition prematurely. Both the WADA and the International Olympic Committee (19) have acknowledged this risk. Consequently, gene doping has been officially classified among the prohibited categories of substances and methods (20).

Bojarczuk (21) states that the exploration of athletic talent through genetics, along with the ethical challenges it presents, has become an increasingly significant and contentious issue within the scientific community. Advances in molecular biology and genetics have deepened our understanding of the biological foundations of athletic performance, offering innovative approaches to talent identification and potential performance enhancement. However, these developments also raise complex ethical concerns related to fairness, equity, and athlete well-being. The convergence of genetics and sport introduces profound ethical dilemmas that demand thoughtful analysis and clear ethical guidance to safeguard the integrity of competition and uphold equal opportunity for all athletes. Debates have emerged around controversial issues such as genetic discrimination, the creation of a genetic underclass among athletes, and the long-term implications for future generations. At the same time, proponents argue that genetic testing could enhance athletic potential and contribute to the advancement of sports and human performance. These conflicting perspectives highlight the urgent need for a comprehensive ethical framework to govern the responsible use and regulation of genetic technologies in the sporting domain (21).

As stated by (22) ethical concerns about gene doping center on health risks from unpredictable effects of gene therapy, violations of athletes' privacy through misuse of genetic information, and challenges to fair competition due to unequal access, potentially creating a divide between “enhanced” and “unenhanced” athletes that threatens the integrity of sport. While some proponents argue that genetic modification could boost athletic performance, increase excitement in sports, and help reduce gender disparities by equalizing physical abilities, these benefits depend on ensuring safety and equitable access. Additionally, current anti-doping policies are insufficient to detect gene doping, prompting calls for organizations like WADA to develop effective monitoring strategies and ethical guidelines to address the complexities introduced by genetic technologies in athletics (22).

## 3 Discussions

### 3.1 Political influence and bioethics

The link between bioethics in sports and medicine is well established and widely acknowledged. Yet the role of international political dynamics in shaping bioethical discourse - particularly in sport - warrants closer examination. Politics does not determine the intrinsic value of sport itself but instead influences the normative frameworks, regulatory architectures, and institutional priorities within which ethical debates and governance decisions are negotiated. Can the bioethical framework governing sport truly be considered neutral, or is it inherently shaped by political agendas, national ideologies, and institutional power structures?

This section explores how bioethics in sport is not merely a matter of science or fairness, but may be deeply intertwined with geopolitical considerations. The author illustrates her assumption through the following causal chain:

(1) Sport becomes a soft power instrument → (2) Nations invest in PEMs to gain a competitive edge → (3) To protect these advantages, they engage in political lobbying or maneuvering to delay or block regulatory restrictions.

1. Sport as a Soft Power Instrument

As defined by Nye in late 1980, “soft power” lies in the ability to attract and persuade. Whereas hard power- the ability to coerce - grows out of a country's military or economic might, soft power arises from the attractiveness of a country's culture, political ideals, and policies (11). In the global political arena, sport serves as a remarkable tool of soft power - a means by which countries enhance their image, assert national identity, and project influence without military or economic coercion.

Hosting major events like the Olympics or FIFA World Cup or achieving international athletic success boosts a nation's global prestige, cultivates national unity, and enhances diplomatic clout.

Success in global sporting events signals national excellence, organizational strength, and even ideological superiority.

Grix and Lee (23) mention the following empirical examples as shown in Table 1:

**Table 1:** International sporting events as instruments of soft power.

Event	Soft power objectives
2008 Beijing Olympic Games (China)	Projected China as a modern, capable, and responsible global power; boosted international image; signaled openness and global leadership.
2010 FIFA World Cup (South Africa)	Showcased Africa's ability to host major events; challenged stereotypes; promoted nation-building and international legitimacy.
2010 Commonwealth Games (India)	Displayed India's organizational capacity and economic growth; reinforced ambition to be seen as a leading international actor.
2014 FIFA World Cup & 2016 Olympic Games (Brazil)	Highlighted Brazil's status as an emerging global player; attracted investment, tourism, and political goodwill.
2014 Winter Olympics & 2018 FIFA World Cup (Russia)	Used mega-events to project soft power, gain global legitimacy, and increase influence in world affairs.
2022 FIFA World Cup (Qatar)	Positioned Qatar as a diplomatic and cultural hub; used sports to enhance global image and influence.

Adapted from Grix & Lee (23).

Hence, to be able to use sport as a soft power, nations begin to prioritize international athletic performance as a strategic goal—not merely for sport's sake, but as part of national diplomacy and image-making.

2. PEMs to Boost National Sport Success

Once sport is politicized as a soft power tool, governments and sporting institutions may turn to PEM's to maximize athlete success. This may include:

State-funded sports science programs, e.g., the Australian Institute of Sport (AIS), founded in 1981 in response to Australia's underwhelming performance at the 1976 Montreal Olympics, integrates disciplines such as sports science, medicine, physiology, nutrition, biomechanics, and psychology to enhance athletic performance. Although the program operates entirely within legal and non-doping frameworks, it exemplifies how government resources can be strategically leveraged to gain a competitive advantage in international sport (24).

Legal, but controversial methods, e.g., altitude simulation or hypoxic chambers are used by athletes from countries like the United States of America, United Kingdom of Great Britain and Northern Ireland, Norway as legal means to increase red blood cell production, mimicking the physiological effects of EPO doping but considered legal by WADA. These methods raise questions about fairness because they provide comparable benefits to banned substances but remain permitted. WADA debated banning them in 2006 but concluded the risk was low; some nations defend these as natural training aids (25, 26).

Gray-zone enhancements that are not yet banned (27), e.g., in 2009, the Fédération Internationale de Natation (FINA) banned the use of polyurethane swimsuits after acknowledging their substantial performance-enhancing effects. However, it did not nullify the records set or revoke the medals won while the suits were permitted, despite clear scientific evidence demonstrating the significant competitive advantage they provided (28).

In some cases, covert or overt doping systems, e.g., between 1968 and the late 1980s, East Germany systematically administered PEDs to approximately 9,000 athletes, achieving significant success in international competitions, especially at the Olympic Games (29); Sochi doping conspiracy: among the most significant findings was the involvement of senior officials - most notably from the Russian Ministry of Sport and the Federal Security Service - in coordinating doping cover-ups, especially during the 2014 Sochi Winter Olympics (30, 31).

While not all PEMs are inherently unethical or illegal, the pressure to win can incentivize national systems to push the boundaries of acceptability - especially when global success is tied to political capital.

3. Political Games to Influence WADA Prohibited List Decisions

If international sporting dominance translates to political capital, sport becomes a proxy battlefield for global reputation. Given this strategic investment in PEMs, countries may engage in political lobbying, behind-the-scenes negotiation or regulatory influence to delay or prevent certain methods from being added to the WADA Prohibited List.

Table 2 presents the framework through which WADA evaluates and determines the inclusion of substances on the Prohibited List. It underscores the complex interplay of political influence and stakeholder interests in shaping these decisions, drawing upon insights from an interview with Dr. Olivier Rabin, Senior Director of Science and Medicine at WADA (32).

**Table 2:** Political and stakeholder influence in WADA's decision-making.

Theme	Relevant statement/observation	How it works
Stakeholder Pressure/Feedback	WADA receives over 100 submissions each year from stakeholders, including national anti-doping agencies, governments, and sport federations. These stakeholders often have opposing views - some strongly supporting inclusion of a substance, others lobbying against it. Comments can be individual or from groups (e.g., Council of Europe). WADA reviews these comments to identify relevant issues.	National lobbying, coordinated comments by groups, advocacy coalitions; stakeholders can push specific agendas.
Geopolitical Influence/Cultural Framing	Medical practice and drug perception differ significantly across countries and cultures. WADA officials explicitly mention that some objections are based more on cultural or national values than on global medical consensus, complicating regulatory decisions. For example, a drug like pseudoephedrine may be sold over-the-counter in one country but restricted in another, leading to conflicting stakeholder lobbying based on local norms. This creates situations where opinions are “polarized” and can be “180 degrees apart."	Balancing cultural/medical variance; differing national/regional perspectives influencing submissions; efforts to unify global standards.
Lobbying *via* Scientific Submissions	Stakeholders are increasingly expected to back their positions with scientific research. However, there's variation in the quality, credibility, and applicability of this evidence. Political interests can shape how such research is framed or presented. WADA experts must weigh the science, recognizing discrepancies or contradictions in research, and do not give equal weight to every comment (e.g., peer-reviewed vs. online articles).	Strategic use of scientific counter-arguments; some anti-doping organizations conduct their own research to influence decisions.
Strategic Delay or Avoidance	Dr. Rabin acknowledges that WADA must resist the natural tendency “to postpone and not to take a decision” amid polarized pressures. This suggests that strong lobbying can at least delay a substance's prohibition pending further review. WADA also justifies delays for “education and adaptation,” which can be a result of, or response to, strong stakeholder resistance.	Deliberate delays and efforts to postpone decisions; negotiations around implementation periods; attempts to stall or manage controversial changes.
Balancing Competing Interests	WADA aims to create a List that is “legally and scientifically accurate” but also serves as an “educational/communication document.” They recognize the complexity and acknowledge they “cannot satisfy everybody” and must rely on “expert judgment to make the most rational decision for the rules to apply globally.” The Executive Committee makes final decisions and can have questions or influence.	Internal WADA process to manage diverse inputs; Executive Committee's role in final approval, where political considerations can be introduced.

LawInSport (32).

To conclude, the ethical challenges posed by PEMs in sport extend far beyond questions of health, fairness and values of olympism - excellence, respect and friendship (19) - they are deeply entwined with geopolitical interests and the soft power ambitions of nations. As sport becomes a vehicle for international prestige, political actors increasingly influence the bioethical frameworks meant to regulate it. As shown above, the regulation of PEMs is not solely a scientific or moral endeavor, but also a politically negotiated process shaped by national priorities, lobbying, and cultural values. Recognizing this intersection is essential for developing a more transparent, equitable, and globally coherent approach to bioethics in sport.

This article does not aim to provide a comprehensive review of all positions in the literature. Instead, it offers a perspective that highlights the intersection of bioethics and politics in sport. A limitation of this approach is its selectivity; further research might systematically analyze how different schools of thought in bioethics, international relations, and sports law converge or conflict. Nevertheless, the perspective advanced here underscores the importance of recognizing that bioethics in sport is not neutral, but politically embedded.

In summary, this article advances the perspective that bioethics in sport is inevitably shaped by the dynamics of soft power and international politics. Looking forward, future work should explore how international institutions can better safeguard ethical principles while acknowledging the realities of geopolitical competition.

## Data Availability

The original contributions presented in the study are included in the article/Supplementary Material, further inquiries can be directed to the corresponding author.
